# The Development of Innovative Handheld Devices to Augment Cardiopulmonary Resuscitation Therapy and External Cardioversion and Defibrillation

**DOI:** 10.19102/icrm.2017.081201

**Published:** 2017-12-15

**Authors:** Melanie L. Gershman, Brandon S. Needelman, Sam N. Schwarzwald, Todd J. Cohen

**Affiliations:** ^1^NYU Winthrop Hospital, Mineola, NY, USA

**Keywords:** Active compression-decompression, cardiopulmonary resuscitation, cardioversion, defibrillation, impedance threshold device

## Abstract

Active compression-decompression (ACD) cardiopulmonary resuscitation (CPR) devices were conceived and invented by Drs. Todd Cohen and Keith Lurie to improve the low survival rates for conventional CPR. Active decompression creates greater chest recoil as compared with the passive decompression used in standard CPR, leading to increased preload and greater cardiac output. ACD CPR devices use a suction cup to adhere themselves to the patient’s chest. Putting downward force onto the device allows for the operator to actively compress the chest. Active chest decompression is achieved by pulling up on the device (using its suction), which in turn pulls up on the patient’s chest, enabling greater chest expansion. Animal and human studies have demonstrated an improvement in overall circulation when using ACD CPR versus standard CPR. The impedance threshold device (ITD) was created to enhance ACD CPR. This device connects to the patient’s airway and prevents the influx of air during chest decompression. ACD CPR plus ITD improves myocardial and cerebral perfusion, increases survival rates, and is associated with more favorable neurological outcomes. CPR guidelines permit the use of the above two devices by properly trained personnel. Additionally, an insulated handheld compression device can be actively applied over a standard defibrillation patch to decrease transthoracic impedance. This methodology has been incorporated into a patented PrestoPush^™^ device (Nexus Control Systems LLC, Port Washington, NY, USA), which is a handheld external cardioversion/defibrillation augmenter. This paper reviews the development of these innovative devices to augment standard CPR and external cardioversion and defibrillation, including summaries of studies the authors consider to those that are most significant in ACD CPR development.

## Introduction

Handheld devices have been developed to augment cardiopulmonary resuscitation (CPR), as well as to improve the efficacy of external cardioversion and defibrillation. This paper describes these innovations, their literature, and recommendations in usage.

### Background

According to the American Heart Association (AHA), over 550,000 people experienced a cardiac arrest in the United States in 2016, with survival rates of 12% and 24.8% for out-of-hospital and in-hospital episodes, respectively.^1^ Since the AHA first endorsed CPR in the 1960s, there have been few updates to the protocol until the last two decades.^2^ In a letter to the editor of the *Journal of the American Medical Association*, Dr. Keith Lurie described a 1985 case in which a toilet plunger was used to aid in the resuscitation of a 65-year-old man with coronary artery disease after traditional CPR failed. It was hypothesized that the plunger provided both active compression and decompression of the chest, and may have assisted with ventilation.^3^ This led Dr. Lurie and Dr. Todd Cohen to create an active compression-decompression (ACD) CPR device.

### The unmet need

There is a need to update standard cardiac arrest CPR methods due to the low survival rate associated with this technique. The goal is to create a device that will augment circulation, increase CPR effectiveness, and improve survival. No specifically designed cardioversion/defibrillation devices exist to control the force applied over an external skin patch to reduce transthoracic impedance and improve procedural efficacy; the proposed device was also designed to fill this void.

### ACD CPR concept

During standard CPR, there is active chest compression where the resuscitator pushes down on the patient’s chest, and passive chest compression as the chest recoils, respectively. In ACD CPR, the decompression becomes active, and the device sucks up on the chest. This provides more pronounced recoil, leading to greater negative pressure within the thorax to enhance cardiopulmonary circulation.^4,5^

Greater chest recoil draws more blood back into the heart, increasing the preload and cardiac output.^6^ The ACD CPR device can also provide deeper and faster chest compressions than its traditional counterpart, which have been associated with improved survival following cardiac arrest.^7^

The handheld ACD CPR device is comprised of a suction cup, handle, and force gauge.^6^ The suction cup is placed on the individual’s chest, where it functions much like a plunger, pulling up on the chest to create active decompression. A compression pad lines the suction cup, acting like a cushion. The handle allows for a transfer of force from the rescuer to the distressed individual.^5^ The gauge on the top of the pump shows the forces applied during compression and decompression, while the audible metronome guides the proper compression rate.^6^ This device requires minimal training and causes less operator fatigue than traditional CPR.^8^

**Figure 1:**
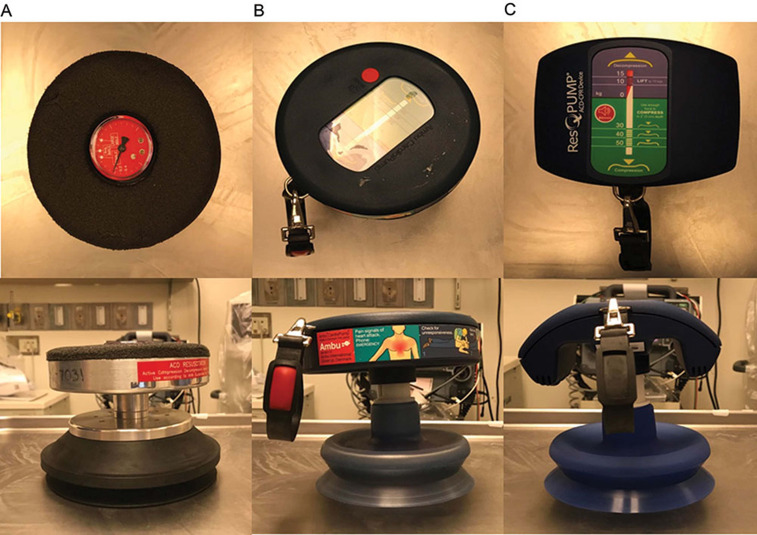
**A:** The first ACD CPR device used in human trials, manufactured by AMBU Inc. (Copenhagen, Denmark). **B:** The ACD CPR device known as the CardioPump^®^ (AMBU Inc., Copenhagen, Denmark). **C:** The ACD CPR device known as the ResQPump^®^ (Zoll Medical Corp., Chelmsford, MA, USA).

**Figure 1** demonstrates the three versions of the device and how it was developed. **Figure 1A** shows the first device used in human trials, manufactured by AMBU Inc. (Copenhagen, Denmark). **Figure 1B** depicts the CardioPump^®^ (AMBU Inc., Copenhagen, Denmark), a lighter and more colorful commercial design that includes a crash cart strap, a more ergonomic handle, a built-in metronome, and embossed instructions for use. This model was used in the first multicenter trial in the early 1990s. **Figure 1C** is the current device, the ResQPump^®^ (Zoll Medical Corp., Chelmsford, MA, USA), which is approved for use by the US Food and Drug Administration (FDA).

## Trials

### Early feasibility

A canine study was published in 1992 by Cohen et al. to compare the efficacies of standard CPR and ACD CPR using a simple handheld suction device planted on the middle of the sternum. After inducing ventricular fibrillation, the eight non-ventilated anesthetized dogs were randomized to receive ACD CPR and standard CPR sequentially, with each technique conducted under the same parameters but in different orders. Transesophageal pulsed wave Doppler echocardiography from the main pulmonary artery revealed that ACD CPR demonstrated significantly improved coronary perfusion pressure, cardiac output, minute ventilation, and systolic arterial pressure as compared with standard CPR.^9^

Later that year, a study conducted by Lindner et al. investigated the effects of ACD CPR and standard CPR on myocardial and cerebral blood flow in pigs. After inducing ventricular fibrillation, 14 pigs were randomized to receive either ACD CPR or standard CPR under the same parameters for five minutes. Following this interval, radiolabeled microspheres were injected into the pigs and showed that ACD CPR yielded a significantly higher median cerebral blood flow and improved aortic, systolic, and diastolic pressures. It also improved coronary systolic and diastolic perfusion pressures, end-tidal carbon dioxide (CO_2_), cerebral oxygen delivery, and cerebral perfusion pressure to greater extents than did standard CPR.^10^

### Human studies

In 1992, Cohen et al. published the first human study that examined the efficacy of ACD CPR using the device shown in **Figure 1A**. Ten patients were randomized to be given two minutes of either standard CPR or ACD CPR, followed by two minutes of the other technique. The authors reported significant increases in blood circulation, end-tidal CO_2_ concentration, and systolic arterial pressure associated with ACD CPR use in comparison with traditional CPR. Furthermore, the improved systolic arterial pressure correlated with increased cerebral perfusion. The authors concluded that cardiovascular, pulmonary, and systemic hemodynamics improved significantly with the use of ACD CPR as opposed to standard CPR.^8^

A 1993 study by Tucker et al. compared transmitral flow and left ventricular volume between ACD CPR and standard CPR.^11^ Five patients were randomized using a methodology similar to that employed by Cohen et al.^7^ Data collected by transesophageal echocardiography revealed that ACD CPR led to increased transmitral flow, end-decompensation left ventricular volume, and stroke volume to greater degrees than did conventional CPR. Unfortunately, no cardiac arrest patient in either group survived.^11^

In a 1993 study published in *The New England Journal of Medicine*, Cohen et al. compared the short-term outcomes of ACD CPR and standard CPR with respect to in-hospital cardiac arrest management. Sixty-two patients who experienced witnessed cardiac arrest were randomly assigned to receive either ACD CPR or standard CPR under identical conditions. This study was terminated by the FDA prior to completion but demonstrated that ACD CPR led to significantly higher initial resuscitation and 24-h survival rates, as well as better neurologic outcomes. Two patients who received ACD CPR survived to hospital discharge, while no patient receiving standard CPR lived to this endpoint.^12^ ACD CPR also increased survival to intensive care unit admission in a 1994 study by Shultz et al., with an increase in favorable neurological outcomes in patients who received ACD CPR as compared with patients who received standard CPR.^13^ In 2016, Gunaydin et al. studied 181 patients who were randomly assigned to receive either standard or ACD CPR. They found no significant differences in survival or discharge rates; however, standard CPR had a higher complication rate, including a higher incidence of rib fractures.^5^

### Flaws with using ACD CPR alone

Multiple studies reported no significant increase in patient survival with ACD CPR in comparison with traditional CPR. In 1999, Maur et al. performed a combined analysis to evaluate the effects of standard and ACD CPR. They found greater rates of one-hour survival with ACD CPR use but no significant difference in survival to discharge.^14^ Notably, one of the included studies used medical personnel who had worked with the ACD CPR device for more than one year,^15^ while practitioners in other investigations had used ACD CPR for only a few weeks prior to the commencement of the respective studies.^14^ Plaisance et al. reported improved survival rates with ACD CPR versus standard CPR techniques, suggesting that familiarity with the ACD CPR device is a possible contributor to increased patient survival rate.^5,14^

A 2013 meta-analysis by Luo et al. analyzed a total of 13 studies and found improved restoration of spontaneous circulation (ROSC) and increased 24-hour survival for ACD CPR versus standard CPR; however, there were no significant differences in survival rates to hospital admission or hospital discharge. The authors concluded that ACD CPR does not provide significant benefits over standard CPR.^16^ Another meta-analysis by Wang et al. suggested that the ACD CPR device is difficult to adhere to the patient’s chest during resuscitation.^17^

### Addition of the impedance threshold device

**Figure 2:**
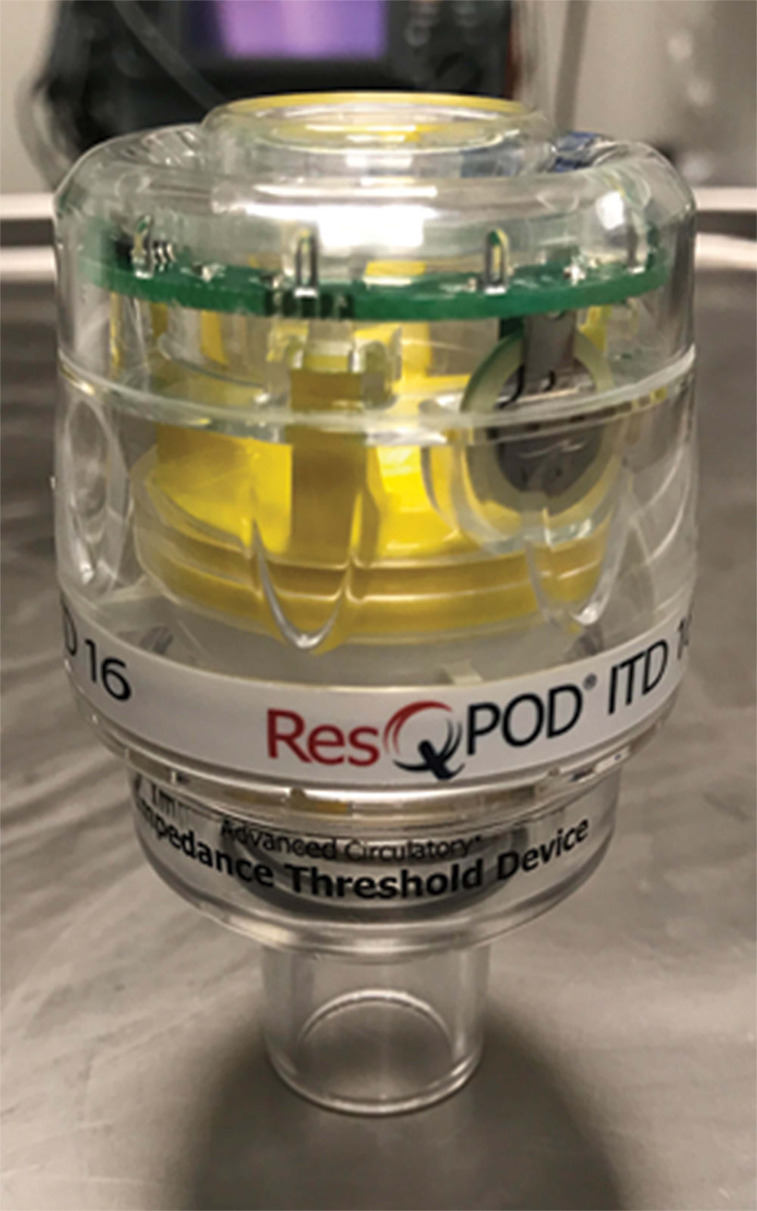
The impedance threshold device known as the ResQPod^®^, initially manufactured by Advanced Circulatory Systems Inc. (Roseville, MN, USA), and now manufactured by Zoll Medical Corp. (Chelmsford, MA, USA).

During a 1994 trial evaluating standard and ACD CPR, the investigators observed that temporary occlusion of the endotracheal tube in one patient during standard CPR produced a more negative intrathoracic pressure than obtained with ACD CPR during active decompression.^18^ These results led to the testing of an impedance threshold device (ITD) called the ResQPod^®^ (Zoll Medical Corp., Chelmsford, MA, USA) along with the ResQPump^®^ (Zoll Medical Corp., Chelmsford, MA, USA), to augment the benefits of ACD CPR.^19^ The ResQPod^®^ (Zoll Medical Corp., Chelmsford, MA, USA) can attach to a facemask or endotracheal tube **(Figure 2)**. An ITD is used during basic and advanced life support as part of the airway, preventing inspiration during the CPR decompression phase. The upward movement of the thoracic wall during this phase leads to a drop in intrathoracic pressure. By preventing air influx, the ITD permits a greater negative pressure to build, allowing for greater cardiac and cerebral perfusion with ACD CPR alone.^6,20–22^

In 2011, the randomized ResQTrial study encompassing 46 emergency medical service agencies was published in *The Lancet*. This study examined the differences between standard CPR and ACD plus ITD CPR (using the ResQPump^®^ **(Figure 1C)** with the ResQPod^®^ **(Figure 2)**; Zoll Medical Corp., Chelmsford, MA, USA) or patients with a suspected non-traumatic out-of-hospital cardiac arrest due to cardiac causes.^6^ The use of an ACD plus ITD CPR device, known as the ResQCPR^™^ system (Zoll Medical Corp., Chelmsford, MA, USA) yielded statistically significant increases in survival to hospital discharge and one-year survival rate over standard CPR. Although the intervention group had an increased incidence of pulmonary edema, there was almost no difference in post-cardiac arrest neurological function in the intervention group versus in the control (standard CPR) group. Relative survival to hospital discharge and one-year survival increased by almost 50% when using ACD plus ITD CPR. This study became the foundation for the FDA’s approval of the ResQCPR^™^ system (Zoll Medical Corp., Chelmsford, MA, USA) in March 2015.^23^ At the time, the original manufacturer of both the ResQPod^®^ and ResQPump^®^, Advanced Circulatory Systems Inc. (Roseville, MN, USA), was acquired by Zoll Medical Corp. (Chelmsford, MA, USA), bringing these two devices under the latter company’s umbrella.

Following completion of the ResQTrial, Frascone et al. assessed whether ACD plus ITD CPR would improve survival following cardiac arrest independent of the out-of-hospital non-traumatic etiology. They found a 38% increase in survival to hospital discharge with favorable neurologic outcomes, and a 39% relative increase in one-year survival following cardiac arrest for ACD plus ITD CPR, independent of the cause of cardiac arrest.^24^ A 2015 review concluded that compared with standard CPR, ACD plus ITD CPR achieved increased blood flow and associated patient survival rates for out-of-hospital cardiac arrest regardless of etiology.^7^

### Mechanical ACD devices

**Figure 3:**
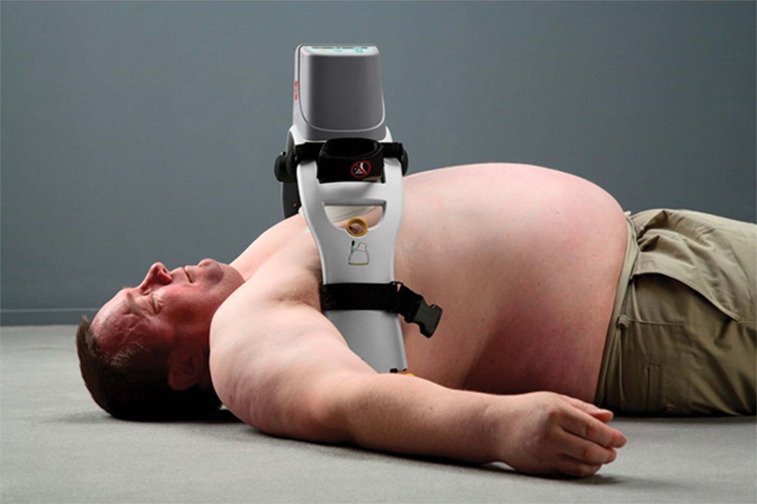
The LUCAS^®^ 3 (Physio-Control, Inc., Redmond, WA), a mechanical ACD CPR device. Photo courtesy of Physio-Control, Inc. ACD: active compression-decompression; CPR: cardiopulmonary resuscitation.

A mechanical version of the ACD device was created and termed the Lund University Cardiopulmonary Assist System (LUCAS^®^) **(Figure 3)**. This gas-driven device provides mechanical compression and active decompression.^25,26^ It is composed of a silicone rubber suction cup with a pneumatic cylinder on a handle connected to a back plate. The LUCAS^®^ allows for more uniform chest compressions compared with conventional CPR.^27^ The machine provides uninterrupted, high-quality chest compressions without human fatigue. Because the LUCAS^®^ does not require medical technicians to utilize their hands to perform chest compressions, these individuals can sit properly strapped into the moving vehicle or focus on performing other lifesaving tasks, including starting an intravenous line, placing an advanced airway, and contacting the hospital to provide updates on the patient’s condition.^27^ The LUCAS^®^ is now in its third generation as the LUCAS^®^ 3 (Physio-Control, Inc., Redmond, WA), which offers improved maintenance and handling, as well as the new feature of wireless access to data collected by the device.^28^

### CPR guidelines

ACD CPR was described in the 2015 CPR and emergency cardiovascular care guidelines as an alternative technique to standard CPR, but the results of its use were mixed.^29^ Multiple studies have shown that ACD CPR increases short-term survival, ROSC, and neurological intactness as compared with standard CPR.^12,15,30–33^ Conversely, other investigations reported no difference in ROSC or survival associated with the use of ACD CPR.^34–40^ Due to the current lack of consensus, the guidelines do not officially recommend for or against ACD CPR use, but do note that the technique can be considered if the user is trained or supervised by trained professionals while using the device.^29^

The guidelines also discuss the use of ACD plus ITD CPR and mention studies with varied results. Some found no difference in survival to discharge between ACD CPR with or without the ITD.^21,22,41^ One trial comparing conventional CPR and ACD plus ITD CPR found increased one-year survival following cardiac arrest with ACD plus ITD CPR use.^8,24^ Due to a lack of research on this method, however, it is not a recommended alternative for conventional CPR unless performed by trained personnel.^29^

Mechanical chest compression piston devices are also mentioned in the guidelines. Two studies conducted using the LUCAS^®^ and three studies that tested other mechanical chest compression devices, respectively, found no difference in patient survival when comparing the use of mechanical and manual chest compression techniques.^42–46^ One other trial using the LUCAS^®^ reported a negative correlation between mechanical ACD CPR and survival with favorable neurologic outcomes.^47^ The guidelines state that the use of mechanical piston devices is a comparable method to the performance of manual CPR when performed by properly trained individuals.^29^ Such devices may enable staff to assist in other aspects of advanced cardiac life support.

### A handheld active compression device to augment external cardioversion and defibrillation

**Figure 4:**
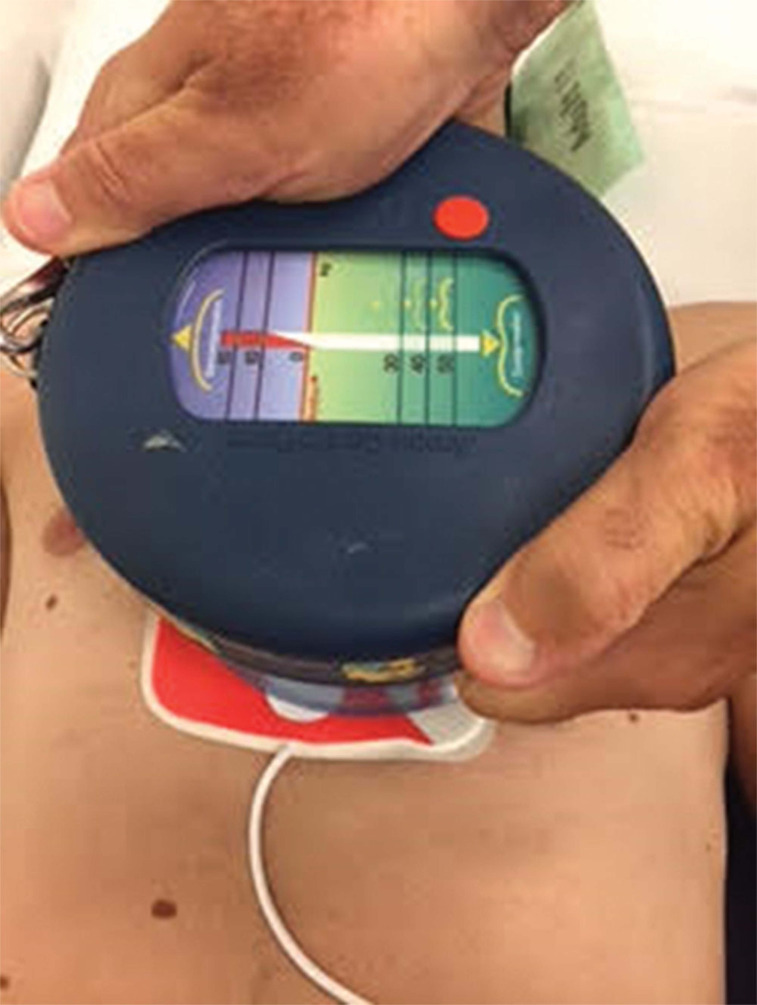
An example of ACD^2^ in which a defibrillation patch is placed under the ACD CPR device.

In 1995, a study described a new technique called ACD defibrillation (ACD^2^), in which a defibrillation skin patch was placed inside the suction cup of an ACD device **(Figure 4)**. By placing the defibrillation pad underneath the device, defibrillation was delivered to the patient during the downstroke of chest compressions during continuous CPR. ACD^2^ was tested on seven canines with induced ventricular fibrillation, yielding a significant reduction in transthoracic impedance as compared with the standard CPR technique. Notably, reduced transthoracic impedance may increase defibrillation efficacy. Although not statistically significant, improvements in defibrillation thresholds were noted.^48^ In 1996, Cohen et al. tested this technique on humans. Standard cardioversion was first attempted in 22 patients with atrial fibrillation; if it failed, active compression cardioversion was used. Four of the five patients who failed initial standard cardioversion were converted to sinus rhythm using active compression cardioversion. This study was the first human trial to demonstrate the successful use of a simple, handheld, insulated device capable of applying calibrated pressures over a cardioversion/defibrillation patch to reduce transthoracic impedance.^49^

**Figure 5:**
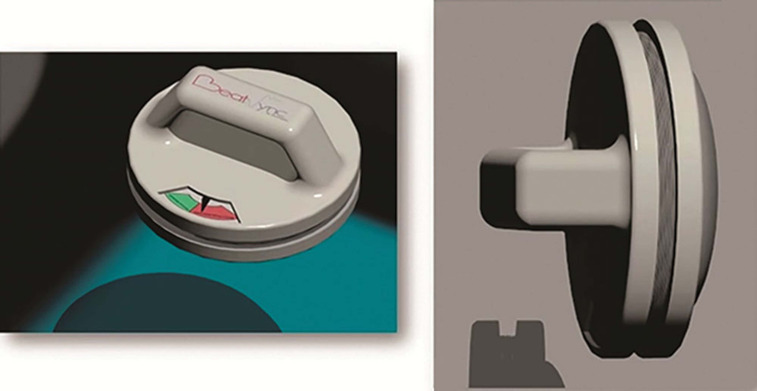
The PrestoPush^™^ (Nexus Control Systems LLC, Port Washington, NY, USA) is a handheld device designed to improve the efficacy of external cardioversion and defibrillation. Image reprinted with permission from: Cohen TC, eds. *Practical Electrophysiology.* 3rd ed. Malvern, PA: HMP Communications; 2016: 17-171.

**Figure 6:**
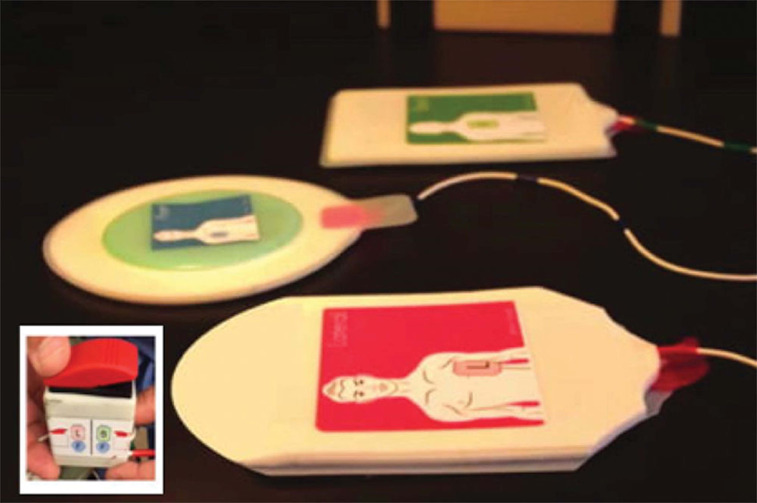
The PrestoPatch^™^ (Nexus Control Systems LLC, Port Washington, NY, USA) consists of an arrangement of three defibrillation patches and a high-voltage switch. Large image reprinted with permission from: Cohen TC, eds. *Practical Electrophysiology.* 3rd ed. Malvern, PA: HMP Communications; 2016: 17-171. The lower left corner insert shows the switch box, which connects to the three-patch system to provide shocks. Insert image added courtesy of Nexus Control Systems, LLC.

In 2013, a team of biomedical engineering students from Johns Hopkins University led and supported by Dr. Cohen adapted ACD^2^ principles to a handheld insulated device called the PrestoPush^™^ (Nexus Control Systems LLC, Port Washington, NY, USA) **(Figure 5)**. This custom-designed, handheld insulated device containing a gauge to control and guide the force applied to the chest was created to improve external cardioversion and defibrillation. This device may increase the effectiveness of external cardioversion and defibrillation by decreasing the intrathoracic impedance, which is useful in patients who are clinically obese.^2^ The same team also developed a vector-switching device called the PrestoPatch^™^ (Nexus Control Systems LLC, Port Washington, NY, USA) **(Figure 6)**. This device consists of three defibrillator patches, along with a high-voltage switch used to alter the shocking vector without moving the patches. In 2013, the Johns Hopkins University team won the National Collegiate Inventors Competition for the PrestoPatch^™^ and PrestoPush^™^ (both Nexus Control Systems LLC, Port Washington, NY, USA).

## Conclusions

Over time, ACD CPR has evolved from its original concept as a handheld plunger appliance to the more sophisticated ResQPump^®^ CPR device (Zoll Medical Corp., Chelmsford, MA, USA) and the LUCAS^®^ mechanical version. Selected studies highlighted in **Table 1** illustrate this transformation. Adjuncts such as the PrestoPatch^™^ and PrestoPush^™^ assistive devices (both Nexus Control Systems LLC, Port Washington, NY, USA) have also been introduced. Despite these significant advances, there are still no strong recommendations for the use of these tools in current CPR guidelines, though they may be considered in scenarios in which providers are adequately trained and monitored in their use. These innovative products are derivatives of active compression and decompression concepts applied to the cardiovascular system. Future methods and devices may potentially be applied to other regions of the body to augment medical treatments.

## Figures and Tables

**Table 1:** Selected studies of ACD CPR

Study	Aim/Goal of Study	Number of Participants	Results/Conclusions
Gunaydin et al.^^5^^	Compare survival and discharge rates in humans with out-ofhospital or in-hospital cardiac arrest.	181	No significant differences in survival or discharge rates were observed. Standard CPR had higher complication rates versus those with ACD CPR.
Aufderheide et al.^6^	Examine differences between standard CPR and ACD CPR plus ITD use in humans with out-ofhospital cardiac arrest.	1,653	ACD CPR plus ITD increased survival to hospital discharge and one-year survival by almost 50% as compared with standard CPR.
Cohen et al.^8^	Examine the efficacy of ACD CPR with standard CPR use in humans.	10	ACD CPR elicited significantly increased blood circulation, end-tidal CO_2_ concentration, and systolic arterial pressure, as well as improved systolic arterial pressure, in comparison with standard CPR.
Cohen et al.^9^	Compare standard CPR with ACD CPR use in dogs.	8	ACD CPR had significantly improved coronary perfusion pressure, cardiac output, minute ventilation, and systolic arterial pressure as compared with standard CPR.
Lindner et al.^10^	Examine the effects of standard CPR with ACD CPR use onmyocardial and cerebral blood flow in pigs.	14	ACD CPR had a significantly higher median cerebral blood flow, aortic systolic and diastolic pressures, calculated coronary systolic and diastolic perfusion pressures, end-tidal CO_2_, cerebral O_2_ delivery, and cerebral perfusion pressure versus standard CPR.
Tucker et al.^11^	Analyze transmitral flow and left ventricular volume for ACD CPR and standard CPR in humans.	5	ACD CPR led to increased transmitral flow, end-decompression left ventricular volume, and stroke volume as compared with standard CPR.
Cohen et al.^12^	Compare short-term outcomes of ACD CPR and standard CPR usefor in-hospital cardiac arrests in humans.	62	ACD CPR showed a significantly higher initial resuscitation rate, 24-hour survival rate, and better neurologic outcomes versus standard CPR. Two patients receiving ACD CPR lived to hospital discharge, while no patient receiving standard CPR lived to this endpoint.
Plaisance et al.^15^	Compare short-term efficacy of ACD CPR and standard CPR use in humans.	512	ACD CPR showed an improved survival rate compared with standard CPR, suggesting that familiarity with ACD CPR device use is a possible contributor to increased survival rate.
Luo et al.^16^	Meta-analysis comparing ROSC and hospital survival and discharge rates.	787	ACD CPR improved ROSC and 24-hour survival rate in comparison with standard CPR, but there was no difference in survival to hospital admission or hospital discharge.
Frascone et al.^24^	Compare survival of patients using ACD CPR plus ITD and standard CPR in humans.	2,738	ACD CPR plus ITD demonstrated significantly increased survival to discharge with favorable neurological outcomes and with respect to one-year survival as compared with standard CPR.
Cohen et al.^48^	Compare ACD^2^ with standard CPR in canines.	7	ACD^2^ had reduced transthoracic impedance and improved defibrillation thresholds as compared with standard CPR.
Cohen et al.^49^	Compare active compression cardioversion with standard cardioversion in humans.	22	First human trial to demonstrate successful use of the ACD^2^ device to reduce transthoracic impedance.

Abbreviations: ACD: active compression-decompression; ACD^2^: active compression-decompression defibrillation; CO_2_: carbon dioxide; CPR: cardiopulmonary resuscitation; ITD: impedance threshold device; O_2_: oxygen; ROSC: restoration of spontaneous circulation.
